# COVID-19 among fit patients with CLL treated with venetoclax-based combinations

**DOI:** 10.1038/s41375-020-0941-7

**Published:** 2020-06-29

**Authors:** Moritz Fürstenau, Petra Langerbeins, Nisha De Silva, Anna Maria Fink, Sandra Robrecht, Julia von Tresckow, Florian Simon, Karin Hohloch, Jolanda Droogendijk, Marjolein van der Klift, Ellen van der Spek, Thomas Illmer, Björn Schöttker, Kirsten Fischer, Clemens M. Wendtner, Eugen Tausch, Stephan Stilgenbauer, Carsten U. Niemann, Michael Gregor, Arnon P. Kater, Michael Hallek, Barbara Eichhorst

**Affiliations:** 1 Department I of Internal Medicine, Center for Integrated Oncology Aachen Bonn Cologne Duesseldorf, German CLL Study Group, University Hospital Cologne, University of Cologne, Cologne, Germany; 2 Department of Internal Medicine, Hematology and Medical Oncology, Kantonsspital Graubünden, Chur, Switzerland; 3 Department of Hematology and Oncology, University Hospital Göttingen, Göttingen, Germany; 4 Division of Hematology and Oncology, Elisabeth-TweeSteden Ziekenhuis, Tilburg, The Netherlands; 5 Division of Hematology and Oncology, Amphia Ziekenhuis Breda, Breda, The Netherlands; 6 Department of Internal Medicine, Ziekenhuis Rijnstate, Arnhem, The Netherlands; 7 BAG Freiberg-Richter, Jacobasch, Wolf, Illmer, Dresden, Germany; 8 Hämatologisch-onkologische Schwerpunktpraxis Würzburg, Würzburg, Germany; 9 Department of Hematology, Oncology, Immunology, Palliative Care, Infectious Diseases and Tropical Medicine, German CLL Study Group, Munich Clinic Schwabing, Munich, Germany; 10 Department of Internal Medicine III, University of Ulm, Ulm, Germany; 11 Department of Internal Medicine I, Saarland University Medical School, Homburg/Saar, Germany; 12 Department of Hematology, Rigshospitalet, Copenhagen University Hospital, Copenhagen, Denmark; 13 Division of Hematology, Cantonal Hospital of Lucerne, Lucerne, Switzerland; 14 Department of Hematology, Cancer Center Amsterdam, Amsterdam University Medical Centers, University of Amsterdam, Amsterdam, The Netherlands; 15 Cologne Excellence Cluster on Cellular Stress Response in Aging-Associated Diseases (CECAD), University of Cologne, Cologne, Germany

**Keywords:** Chronic lymphocytic leukaemia, Randomized controlled trials, Infectious diseases, Targeted therapies

## To the Editor:

With more than 5 million proven infections and more than 300,000 associated deaths worldwide [1], the SARS-CoV-2 pandemic poses unprecedented challenges to health-care professionals and especially those treating and caring for patients with malignant hematological diseases. These patients often have multiple different risk factors for severe infections [2]. Chronic lymphocytic leukemia (CLL) is the most common form of leukemia and infections are a known contributor to morbidity and mortality due to a disease-inherent immunodeficiency [3, 4]. Considering this multifactorial immune defect, it appears conceivable that patients with CLL are more susceptible to infections with SARS-CoV-2 and more likely to develop severe courses of the associated respiratory disease COVID-19, especially when under additional immunosuppression by chemoimmunotherapy (CIT). Few case reports on COVID-19 in CLL patients from countries with suspected different prevalence rates of COVID-19 have been published so far. The publications report a patient after first-line treatment with single-agent chlorambucil, a case series with four treatment-naive CLL patients, a case series of eight patients on Bruton tyrosine kinase (BTK) inhibitors and most recently a heterogeneously treated population of four patients from the Hospital Clinic of Barcelona [5–8]. While it has been hypothesized that the BTK inhibitor ibrutinib might have protective effects against COVID-19 by attenuating hyperinflammatory responses, there is currently no data on COVID-19 in patients receiving venetoclax-based treatments [7, 9]. A recent study has suggested a reduction of CLL-inherent immunosuppression after successful treatment with venetoclax-based regimens [10]. In light of these data we sought to determine the incidence, severity, and possible risk factors of COVID-19 cases in a well-defined cohort of patients with CLL receiving venetoclax-based combination treatments as first-line therapy in a prospective clinical trial.

The GAIA/CLL13 trial (NCT02950051) is a multicenter phase 3 investigator-initiated trial with sites in nine European countries plus Israel. From December 2016 to September 2019, 926 physically fit and treatment-naive patients were randomized into four treatment arms. In the standard arm, CIT with fludarabine, cyclophosphamide plus rituximab (FCR, patients ≤ 65 years) or bendamustine plus rituximab (BR, patients > 65 years) is administered. In the experimental arms 12 cycles of venetoclax-containing regimes are tested: venetoclax plus rituximab (RVe), venetoclax plus obinutuzumab (GVe) and venetoclax plus ibrutinib and obinutuzumab (GIVe). Patients with del(17p) or *TP53* mutation were not eligible.

Between March and April 2020 seven patients within the GAIA/CLL13 trial developed COVID-19, one in the CIT arm and six patients in the experimental treatment arms (Table 1). The baseline characteristics show a median age of 61 years (range 52–78) and in accordance with the inclusion criteria of the study only few comorbidities and no *TP53* aberrations were documented. All but one patient had completed study treatment at the time point of COVID-19 diagnosis with a median time after end of treatment of 22 (range 1–30) months. All seven patients were tested positive for SARS-CoV-2 by PCR collected from nasopharyngeal swabs. While one patient was isolated in home quarantine, six of seven patients (85.7%) had to be hospitalized and two (28.6%) required treatment on an intensive care unit (ICU), only one patient required invasive mechanical ventilation (Table 1). Two patients died as a result of their SARS-CoV-2 infection, one 58-year-old patient (patient 4) after a prolonged treatment with mechanical ventilation (52 days) on an ICU and a 78-year-old patient (patient 7) who decided against ICU treatment and was treated with best supportive care.

**Table 1:** Patient and treatment characteristics.

	Patient 1	Patient 2	Patient 3	Patient 4	Patient 5	Patient 6	Patient 7
Baseline characteristics							
Age, years	52	60	68	58	63	61	78
Sex	M	F	M	M	M	F	F
Country	GER	NL	NL	CH	GER	GER	NL
CLL characteristics							
Treatment	RVe	RVe	GIVe	GIVe	GVe	FCR	GVe
Date of CLL diagnosis	07/2013	02/2013	03/2018	08/2018	03/2017	07/2011	09/2011
Time from CLL diagnosis to treatment, months	43	56	7	6	1	70	96
Binet stage at screening	B	A	B	B	C	B	A
FISH at screening							
Del(11q)	N	Y	Y	N	N	N	N
Trisomy 12	N	N	N	N	N	N	N
Del(13q)	Y	N	N	N	Y	Y	Y
IGHV mutational status at screening	Unmut.	Unmut.	Unmut.	Mut.	Unmut.	Mut.	Mut.
Comorbidities at screening							
Cumulative Ilness Rating Scale (CIRS) score	5	4	2	0	2	3	2
Hypertension	N	Y	Y	N	N	Y	N
Diabetes	N	N	N	N	N	N	N
Asthma	Y	N	N	N	N	N	N
COPD	N	N	N	N	N	N	N
Cardiovascular Diseases	N	N	N	N	Y	N	N
Obesity (BMI ≥ 30 kg/m²)	N	N	N	N	N	N	N
COVID-19 presentation							
Symptoms at first presentation							
Fever	Y	Y	N	N	N	N	Y
Cough	Y	N	Y	Y	N	Y	Y
Fatigue	N	Y	N	Y	N	Y	N
Dyspnea	N	Y	N	Y	N	N	N
Sore throat	N	N	Y	N	N	N	N
Rhinitis	N	N	N	N	Y	N	N
Anosmia/Ageusia	N	N	N	N	N	N	N
Bilateral pulmonary infiltrates on CT/X-ray	Y	Y	Y	Y	ND	Y	Y
Time EOT to COVID-19, months	26	18	3	1	27	30	NA
COVID-19 treatment and outcome							
Hospitalization	Y	Y	Y	Y	N	Y	Y
Duration of stay, days	16	12	6	55	NA	12	14
Oxygen support	Y	Y	Y	Y	N	N	Y
Type of ventilation/support	HFNC	NC	NC	TI	None	None	NC
Outcome	Resolved	Resolved	Resolved	Death	Resolved	Resolved	Death

*Y* yes, *N* no, *NA* not applicable, *ND* not done. *M* male, *F* female. *GER* Germany, *NL* The Netherlands, *CH* Switzerland. *RVe* rituximab, venetoclax, *GIVe* obinutuzumab, ibrutinib, venetoclax, *GVe* obinutuzumab, venetoclax, *FCR* fludarabine, cyclophosphamide, rituximab. *Unmut.* unmutated, *Mut.* mutated. *HFNC* high flow nasal cannula, *NC* nasal cannula, *TI* tracheal intubation.

**Fig. 1:**
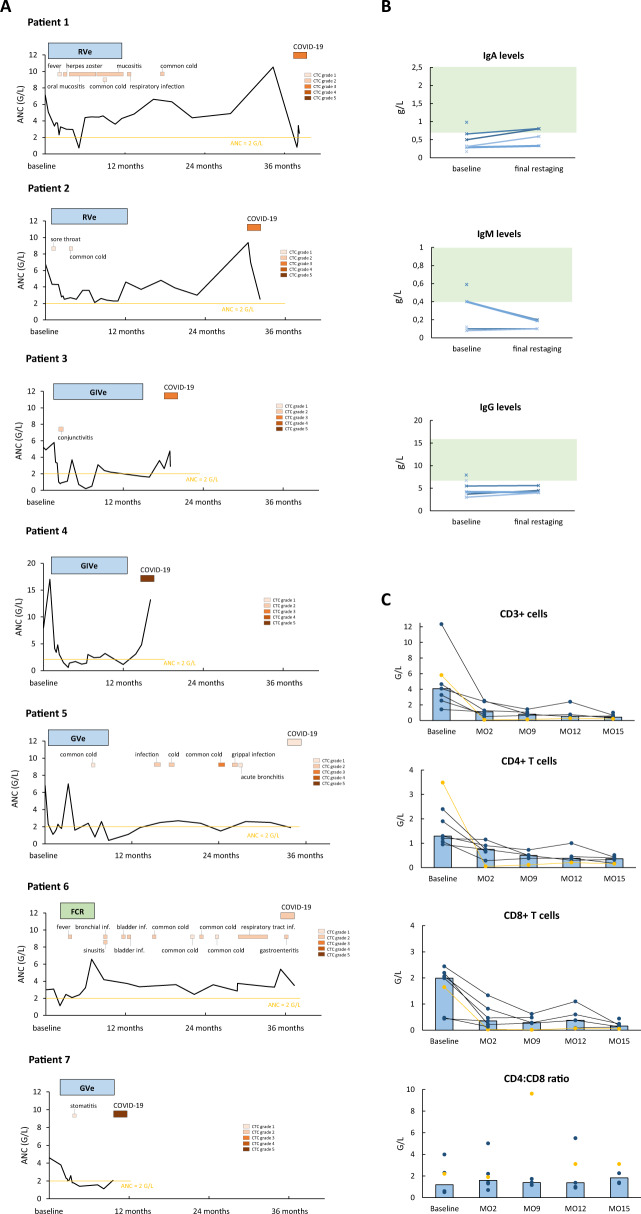
Individual treatment courses and parameters of immune function. **a** The vertical axis represents absolute neutrophil counts (ANC), blue boxes show treatment regimen and duration (RVe rituximab, venetoclax, GVe obinutuzumab, venetoclax, GIVe obinutuzumab, ibrutinib, venetoclax, FCR fludarabine, cyclophosphamide, rituximab). Infections after study inclusion and onset of COVID-19 are depicted in orange boxes. The threshold for neutropenia is shown in yellow. **b** The vertical axis shows levels of immunoglobulins before (baseline) and after treatment (final restaging), normal ranges are indicated in green. Each cross/line represents one patient. **c** Changes in T-cell subpopulations in the course of first-line treatment. Patients on venetoclax combinations are depicted in black, the patient on FCR is shown in orange. Blue bars represent median values of all analyzed patients at each time point.

We assessed different surrogate markers for immune function to elucidate the mechanisms of susceptibility to COVID-19 in our patient cohort (Fig. 1). The frequency of infections observed after start of study treatment differed strongly between the patients. Three patients had a history of multiple (≥2) infections per year since start of study treatment and six of seven (85.7%) patients had at least one episode of neutropenia (Fig. 1a). An analysis of immunoglobulin levels before and after study treatment revealed a substantial humoral immune deficiency with abnormal pretreatment IgG levels in six of seven patients (85.7%) (Fig. 1b). In line with previous data, we show a decrease of the initially expanded CD3+, CD4+ and CD8+ T-cell populations in the course of study treatment (Fig. 1c).

Between March and April 2020, we observed seven cases of COVID-19 among 926 patients in our phase 3 GAIA/CLL13 trial. The estimated cumulative incidence of 755.9 COVID-19 cases per 100,000 persons appears high when compared to age-specific (60–79 years) incidence rates, for instance in Germany (female: 169.5; male: 209) [11]. We also observed a substantially higher hospitalization rate of 85.7% in our patients compared to a study that estimated patients requiring hospitalization at 11.8% (60–69 years) and 16.6% (70–79 years), respectively [12].

This difference is likely due to the multifactorial immune suppression in our patients (Fig. 1). Besides an increased frequency of infections in some and CLL-associated hypogammaglobulinaemia in most patients we also found reduced CD4+ and CD8+ T-cell subpopulations. Adding to this quantitative cellular immune deficiency, T cells are known to be functionally impaired in CLL [13]. In COVID-19, decreased levels of CD4+ and CD8+ T cells were associated with more severe disease courses, suggesting that pre-existing cellular defects might lead to an impaired T-cell response in infected individuals with CLL [14]. Furthermore, in this relatively small case series, the most severe respiratory failures were observed in patients who were still under treatment (patient 7) or had stopped treatment 2 months before (patient 4), which might reflect more severe immune deficiency during ongoing combination treatment. However, the comparably high incidence and hospitalization rate could also reflect a more stringent observation and precautious hospitalization of these patients treated within a clinical trial, though none of the patients had an asymptomatic SARS-CoV-2 infection and the number of unknown cases could be even higher.

Despite the high hospitalization rate, the here observed case fatality rate of 28.6% is similar to the recently published cohort of BTK inhibitor-treated patients with CLL (25%) and lower than in the case series of four treatment-naive patients, of which three had a fatal outcome (75%) [6, 7]. The different cases fatality rates observed between the treatment-naive cohort and our study are most likely due to the different ages of the analyzed populations. All patients with fatal COVID-19 courses described in the publication by Paneesha et al. were between 79 and 81 years of age compared to a median age of 61 years in our cohort. Furthermore, our study population comprises of comparably fit CLL patients with few comorbidities (median CIRS score: 2 [range 0-5]). However, five of our patients had additional risk factors (hypertension, chronic respiratory diseases, cardiovascular disease) for severe COVID-19 as established by recent meta-analyses [15].

To our knowledge, we here report the first analysis of COVID-19 in CLL patients receiving venetoclax-based combinations and CIT as first-line treatment within a large randomized controlled trial. This analysis suggests an increased rate of COVID-19 as well as an increased hospitalization rate in fit patients with CLL. Despite their various CLL-associated immune defects, the majority of patients recovered from COVID-19. As this is an ongoing clinical trial, a benefit-risk assessment is continuously performed by an independent data and safety monitoring board (DSMB). At this time the DSMB had no objection against continuation of the trial or subjects to continue treatment as allocated.
